# Successful extravascular implantable cardioverter-defibrillator implantation in a patient with a preexisting transvenous dual-chamber pacemaker: A case report

**DOI:** 10.1016/j.hrcr.2025.10.025

**Published:** 2025-10-23

**Authors:** Kenneth Kay Leong Khoo, Kit Hou Chiang, Lai Kuan Leong, Kok Choon Cheah, Tiong Kiam Ong, Keng Tat Koh

**Affiliations:** Department of Cardiology, Pusat Jantung Sarawak, Sarawak, Malaysia

**Keywords:** Extravascular implantable cardioverter-defibrillator, P-wave oversensing, Atrial oversensing, End-stage renal disease, Ventricular tachycardia, Dual-chamber permanent pacemaker, Atrial pacing–dependent, Case report

**Key Teaching Points**

• Extravascular (EV) implantable cardioverter-defibrillator implantation can be performed safely and effectively in patients with existing pacemakers, end-stage renal disease, and abandoned transvenous leads. This approach helps mitigate the risks of infection and vascular complications in patients with complex device-related histories.
• Imaging-guided Epsila EV lead placement may be necessary to reduce the risk of P-wave oversensing. Preoperative imaging, such as chest computed tomography scan, can inform individualized lead positioning strategies.
• Tailored device programming, including adjustments to sensitivity settings and blanking periods, is essential to prevent oversensing and inappropriate therapy in patients with existing pacing systems. Intraoperative testing under maximal pacing conditions should be performed to confirm appropriate sensing behavior before finalizing lead placement.

## Introduction

The extravascular implantable cardioverter-defibrillator (EV-ICD) is a novel device that provides pause-prevention pacing, antitachycardia pacing, and defibrillation, while avoiding the potential complications of a transvenous implantable cardioverter-defibrillator (ICD), such as pneumothorax, vascular injury, venous obstruction, and bloodstream infection.^1^^,^^2^ However, EV-ICD implantation is contraindicated in patients with preexisting devices that provide unipolar, dual-chamber, or triple-chamber pacing.^3^ Case reports have described the off-label concomitant use of EV-ICDs and permanent pacemakers, although these involved implanting the EV-ICD first, followed by the pacemaker.

We present a case of successful EV-ICD implantation in a patient with a long-standing, atrial pacing–dependent transvenous dual-chamber permanent pacemaker (TV-DCPPM) implanted for sick sinus syndrome.

## Case report

A 74-year-old woman with underlying diabetes mellitus, hypertension, and gout underwent TV-DCPPM implantation in 1998 for sick sinus syndrome. Two years later, she underwent generator replacement and implantation of a new ventricular lead owing to premature battery depletion. The depletion was attributed to ventricular overpacing at high pacing thresholds resulting from ventricular undersensing. Notably, the old ventricular lead, which was a passive fixation lead, was not extracted and was instead abandoned in situ, whereas the newly implanted ventricular lead was an active fixation type.

In 2022, she was diagnosed as having end-stage renal disease (ESRD) and commenced regular hemodialysis via a right upper limb arteriovenous fistula. The fistula subsequently failed and her dialysis access was converted to a permanent dialysis catheter on the right subclavian vein.

In 2023, she had her third pulse generator box change owing to recommended replacement indicator. Over the years, device interrogations showed that she was atrial pacing dependent (>90%) with occasional ventricular pacing (<0.1%). The atrial lead parameters were normal (impedance 380 Ω; sensing 1.9 mV; bipolar threshold 0.75 V at 0.4 ms) but the ventricular lead parameters showed an elevated, but acceptable pacing threshold (impedance 494 Ω; sensing 7.1 mV; bipolar threshold 1.75 V at 1.0 ms).

**Figure 1:**
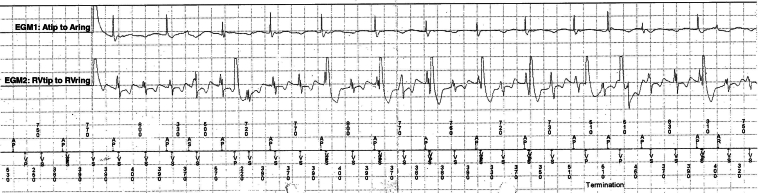
EGM of the transvenous dual-chamber permanent pacemaker showing more rapid ventricular signals than atrial signals and atrioventricular dissociation. EGM = electrogram.

In December 2024, the patient had syncope that resulted in a traumatic subdural hemorrhage. Device interrogation at the time revealed atrioventricular (AV) dissociation with rapid, regular ventricular sensed signals (tachycardia cycle length 320–400 ms), exceeding the rate of atrial pacing (pacing cycle length 720–830 ms) (Figure 1). These findings were consistent with sustained ventricular tachycardia (VT) and correlated with the timing of her syncopal event.

Physical examination revealed dilated veins over her left chest wall and upper limb (ipsilateral to the site of the TV-DCPPM), suggesting the possibility of a chronic left subclavian venous occlusion. Blood tests excluded reversible metabolic causes of VT. Transthoracic echocardiography demonstrated normal valves, preserved ventricular ejection fraction, no regional wall motion abnormalities, and no features of hypertrophic cardiomyopathy. Coronary angiography showed normal coronary arteries. Cardiac magnetic resonance imaging was considered to further evaluate the etiology of VT; however, a shared decision with the patient was made to defer it owing to the rare but serious risk of nephrogenic systemic fibrosis.

Clues to the cause of VT were limited, given that it was recorded only once by her pacemaker and her electrocardiogram was normal. Electrophysiology studies were offered but declined after a thorough discussion of risks, potential findings, and management options. Given her sustained VT causing syncope, an ICD was indicated. However, owing to ESRD with limited vascular access, possible left subclavian occlusion, an abandoned 27-year-old passive lead, a 25-year-old active lead with high thresholds, and a recent generator change, transvenous ICD with lead extraction was not pursued. A subcutaneous ICD was considered but deemed unsuitable, given that the patient had slow VT at 150 beats/min (bpm), which was below the lowest detection zone (170 bpm) of a subcutaneous ICD.^4^ Therefore, EV-ICD implantation was considered the most appropriate option.

**Figure 2:**
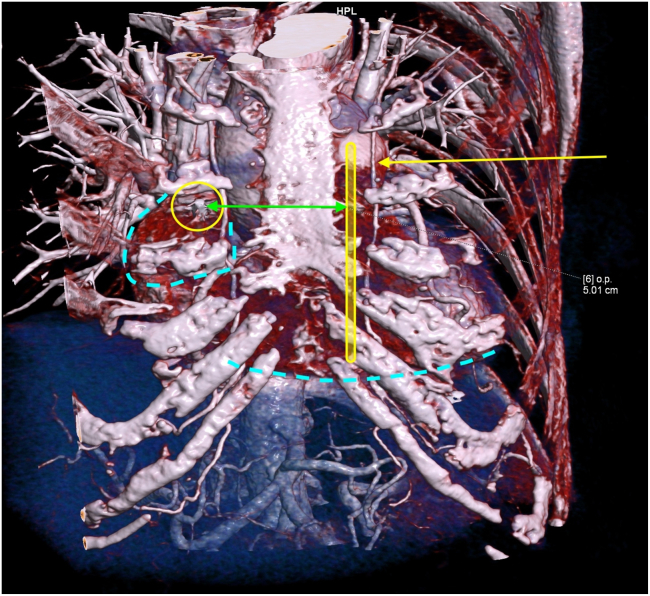
3-dimensional reconstruction of the chest computed tomography. *Yellow rectangle:* Planned Epsila EV lead placement. *Yellow circle:* Atrial lead tip of the transvenous dual-chamber permanent pacemaker. *Yellow arrow:* Left internal mammary artery. *Green arrow:* Distance between the atrial lead tip and the Epsila EV lead, 5.01 cm. *Dotted blue lines:* Right atrial appendage over the right side and the inferior border of the right ventricle. EV = extravascular.

**Figure 3:**
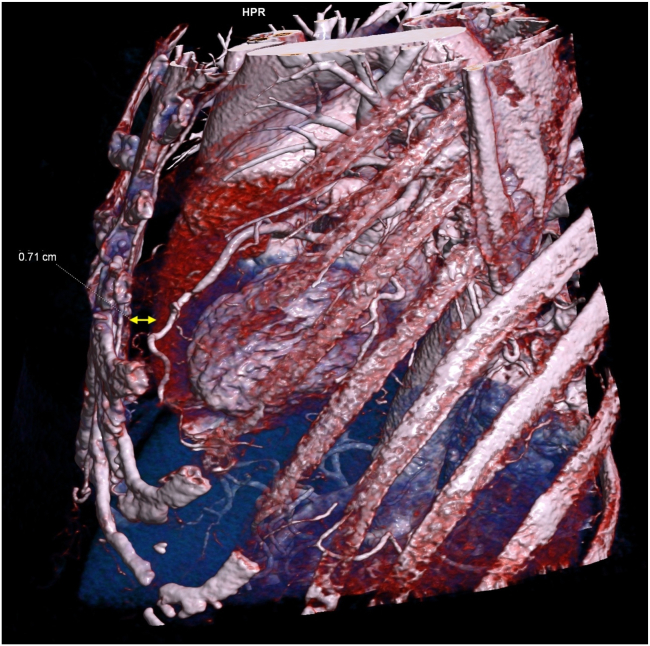
Left lateral view of chest computed tomography with 3-dimensional reconstruction. *Yellow arrow:* Narrowest distance between the sternum and anterior surface of the heart, 0.71 cm.

**Figure 4:**
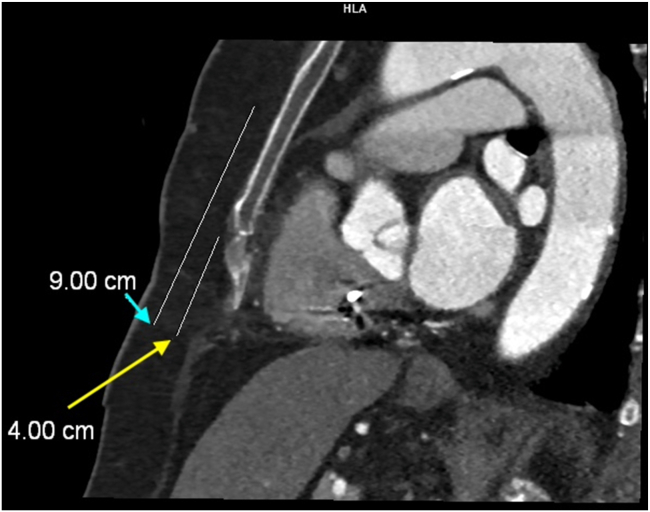
Left lateral view of chest computed tomography. *Yellow arrow:* Distance between the xiphoid process to the xiphisternal junction, 4 cm. Ring 2 of the Epsila extravascular lead was planned to position at 1–2 cm caudal to the xiphisternal junction. *Blue arrow:* Distance between the xiphoid process to the level of the tracheal carina, 9 cm.

Preprocedural chest computed tomography (CT) was performed to understand the anatomy and plan the defibrillator lead positioning of the EV-ICD. The chest CT showed that the tip of the atrial lead was located at the base of the right atrial appendage, along the right parasternal border (Figure 2). Therefore, to prevent oversensing of the atrial pacing spike, the substernal EV-ICD defibrillation lead (Epsila EV, Medtronic) had to be placed in the left para-sternum, medial to the left internal mammary artery. The defibrillation lead also had to be positioned more caudally in relation to the tracheal bifurcation and xiphisternal junction, to ensure that ring 1 and ring 2 were located more caudally and away from the atrial lead tip (Figures 3 and 4).

**Figure 5:**
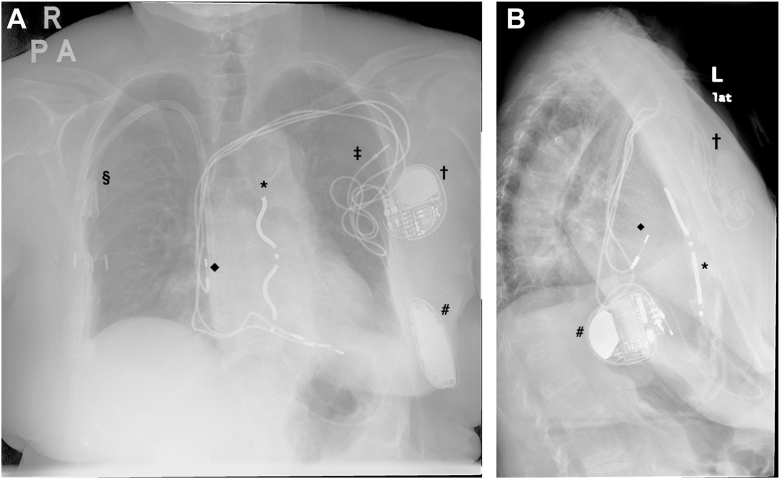
Post-EV-ICD implant chest radiograph. Posteroanterior (A) and lateral view (B) showing the position of the atrial lead of the TV-DCPPM in relation to the Epsila EV lead. ∗ = Epsila EV lead. # = EV-ICD generator. † = TV-DCPPM generator. ‡ = abandoned ventricular lead of TV-DCPPM. ◆ = atrial lead of TV-DCPPM. § = permanent dialysis catheter. EV = extravascular; EV-ICD = extravascular implantable cardioverter-defibrillator; TV-DCPPM = transvenous dual-chamber permanent pacemaker.

**Figure 6:**
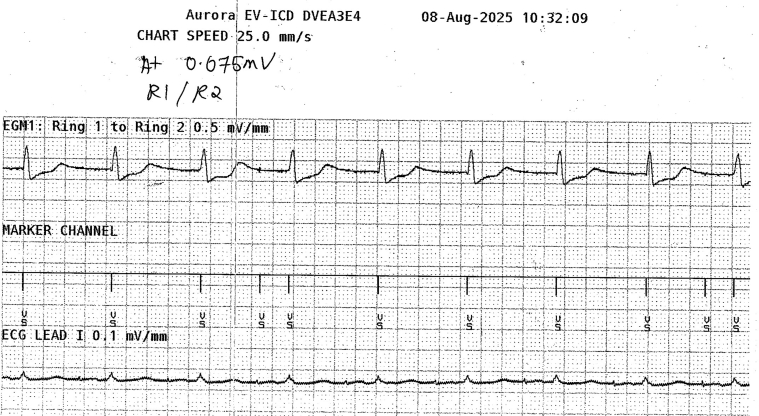
EGM of the extravascular implantable cardioverter-defibrillator during high-output atrial pacing (5 V at 1 ms, bipolar), with sensitivity set at 0.075 mV, and sensing threshold drop time of 500 ms. Atrial oversensing was intermittently detected. ECG = electrocardiogram; EGM = electrogram.

**Figure 7:**
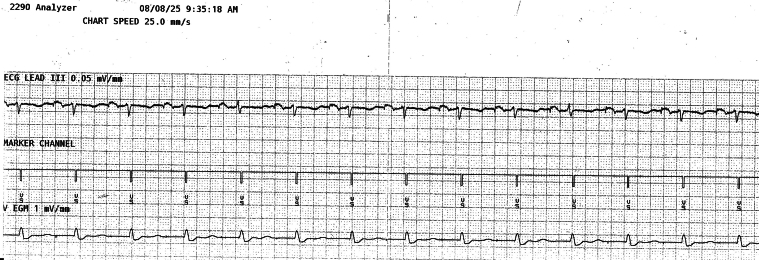
EGM of the extravascular implantable cardioverter-defibrillator during high-output atrial pacing (5 V at 1 ms, bipolar), with sensitivity set at 0.075 mV, and sensing threshold drop time set at 1500 ms. No atrial oversensing. ECG = electrocardiogram; EGM = electrogram.

The EV-ICD (Aurora EV-ICD, Medtronic) system was implanted under general anesthesia on the 8 August 2025. The Epsila EV lead was placed in the substernal space and at the left parasternal position, with the ring 2 at 1 cm caudal to the xiphisternal junction, whereas the EV-ICD generator was positioned in a subcutaneous pocket over the left lateral thoracic wall (Figure 5). Device testing showed a ventricular sensing of 1.9–2.7 mV, pacing threshold of 7 V at 2 ms (rings 1–2), and an impedance of 532 Ω. High-output atrial pacing (5 V at 1 ms, bipolar) from the TV-DCPPM demonstrated intermittent atrial oversensing when the sensitivity was set at 0.075 mV and the sensing threshold drop time was 500 ms (Figure 6). However, after adjusting the sensing threshold drop time to 1500 ms, atrial oversensing was not observed in any of the 3 vectors of the EV-ICD system. Defibrillation threshold (DFT) testing was performed and was successful on single attempt at 40 J. Post-DFT high-output atrial pacing again confirmed the absence of atrial oversensing (Figure 7).

The EV-ICD parameters were finally programmed at a sensitivity of 0.15 mV, postsense blanking period of 150 ms, sensing threshold decay delay of 360 ms, and sensing threshold drop time of 1500 ms. Therapy zones were configured with VF/VT2 zone of >188 bpm, VT1 zone of >146 bpm, and monitoring zone of >136 bpm. The TV-DCPPM was set to an upper tracking rate of 120 bpm and a base rate of 50 bpm. Rate hysteresis mode and rate-adaptive AV were turned off. Pacing output was set at 2.5 V at 0.4 ms (bipolar) for the atrium and 2.25 V at 1 ms (bipolar) for the ventricle.

**Figure 8:**
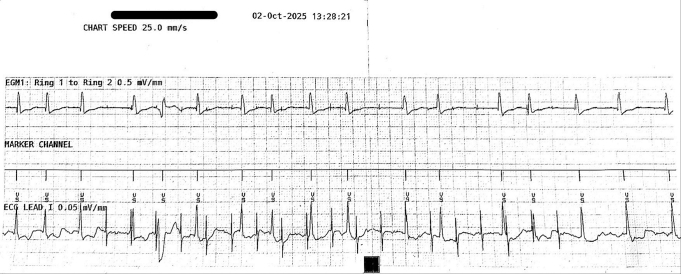
EGM of the extravascular implantable cardioverter-defibrillator during follow-up. High-output atrial pacing (5 V at 1 ms, bipolar) with sensitivity set at 0.075 mV, and sensing threshold drop time set at 1500 ms whereas intrinsic rhythm was atrial fibrillation. No atrial oversensing. ECG = electrocardiogram; EGM = electrogram.

At device interrogation 2 months after implant, she was in atrial fibrillation with an average ventricular rate of 110 bpm. Repeat testing revealed no atrial oversensing during intrinsic rhythm or high-output atrial pacing (Figure 8).

## Discussion

This case highlights the successful implantation of an EV-ICD in an ESRD patient with a long-standing atrial pacing–dependent TV-DCPPM and a recent episode of sustained VT. The decision to implant an EV-ICD was driven by multiple complicating factors, including ESRD with limited vascular access for dialysis, possible left subclavian venous occlusion, presence of a long-standing abandoned passive fixation ventricular lead, long-standing active ventricular lead with increased pacing threshold, and a recent pulse generator change. These factors collectively posed a higher risk of lead extraction, infection, and vascular complications. These risks were mitigated by opting for an EV-ICD. To the best of our knowledge, this represents the first reported case of successful EV-ICD implantation in a patient with a preexisting atrial pacing–dependent TV-DCPPM.

In patients with intrinsic sinus rhythm, the EV-ICD may oversense native P waves, leading to double counting and potentially inappropriate therapies.^2^ Therefore, a key consideration in this case was the potential for device-device interaction between the existing TV-DCPPM and the newly implanted EV-ICD. Given that the patient was atrial pacing dependent, there was a risk that the EV-ICD could potentially oversense the atrial pacing spike, resulting in double counting and inappropriate tachycardia therapy. This concern is relevant because of the anatomic proximity of the Epsila EV lead, which is placed in the substernal space, to the right atrial appendage, where the atrial lead of a transvenous pacemaker is typically positioned.

Römers et al^5^ reported a case of successful TV-DCPPM implantation in a patient with a preexisting EV-ICD, without any adverse interactions. Drawing from that experience, we decided to proceed with EV-ICD implantation in this patient. However, implanting an EV-ICD after a TV-DCPPM is more challenging, because there is limited maneuverable space in the substernal area for the placement of the Epsila EV lead to avoid atrial oversensing. Unlike in the reverse order, where the atrial lead can be actively fixated at alternative sites within the right atrium, away from the substernal Epsila EV lead. Given that the patient was predominantly atrial pacing, we undertook meticulous preoperative planning, as well as intraoperative and postprocedural testing and adjustments, to overcome atrial oversensing.

Preoperative planning included assessment of TV-PPM parameters and evaluation of substernal anatomy relative to the heart chambers and pacing leads using chest CT imaging. Although the patient’s ventricular lead threshold was relatively high (1.0 V at 1 ms), oversensing of ventricular pacing artifacts was not a major concern in this patient. This was because the patient’s ventricular pacing burden was minimal (<0.1%), and during atrial pacing, 1:1 AV conduction was consistently observed without evidence of AV block even up to rates of 120 bpm. This suggested that the need for high-rate ventricular pacing was unlikely. In addition, to mitigate any theoretical risk of the pacemaker delivering high-rate ventricular pacing that might be sensed by the EV-ICD as VT, the pacemaker’s upper sensing and tracking rate was set at 120 bpm. Should the patient develop any atrial tachyarrhythmias in the future, the 120 bpm upper sensing and tracking rate limit would prevent high-rate ventricular pacing that could cause pacemaker–ICD interaction. However, the primary concern was atrial oversensing by the EV-ICD, which may result in double counting and inappropriate therapy, particularly in an atrial pacing–dependent patient. Although the pacemaker was programmed with a stable atrial output of 2.5 V at 0.4 ms, testing for atrial oversensing was also performed at higher outputs during EV-ICD implantation.

The next step involved planning the Epsila EV lead placement using chest CT imaging. Given the position of the atrial lead tip, we identified the left substernal space, medial to the left internal mammary artery and in a more caudal location, which provided the greatest distance from the right atrial appendage and atrial lead tip (Figure 2, Figure 3, Figure 4) to the planned location of the Epsila EV lead. Although we acknowledge that this modified position does not follow standard recommendations, increasing the distance between the atrial lead tip and the Epsila EV lead rings (ring 1 and ring 2) intuitively reduces the likelihood of atrial oversensing.

With this anatomic information in mind, markings were made under fluoroscopic guidance before EV-ICD implantation. In particular, the tracheal bifurcation and xiphisternal junction were identified, and the lead was positioned at the left parasternal junction. Instead of the recommended location of 1 cm above the xiphisternal junction, the ring 2 was placed more caudally, at the level of 1 cm caudal to the xiphisternal junction. Before finalizing the lead position, the EV-ICD system was tested for atrial oversensing across all sensing vectors. These included high-output atrial pacing and evaluation of the EV-ICD’s sensing behavior at higher sensitivity (0.075 mV) with a sensing threshold drop time of 500 ms. After DFT testing, atrial oversensing was reassess with the above settings before concluding the procedure. These strategies ensured sufficient margin of safety when the EV-ICD was finally set at the default sensitivity of 0.15 mV and sensing threshold drop time of 1500 ms.

DFT testing was performed at high energy (40 J) for 2 reasons: to minimize the number of DFT tests in an elderly patient with multiple comorbidities and rule out pacemaker malfunction after high-energy ICD shock. No ventricular pause was observed after the defibrillation with 40 J; therefore, no conflict was observed between the pause-dependent pacing from EV-ICD and the bradycardia pacing from the TV-DCPPM. Given that the base rate of the pacemaker was set at 50 bpm, a sensing threshold drop time of 1500 ms (equivalent to 40 bpm) ensured that the device maintained a lower sensitivity threshold throughout the entire atrial pacing interval, minimizing the risk of atrial oversensing. The rate hysteresis and sleep mode were turned off to maintain a stable base rate.

Looking ahead, if atrial lead failure were to occur, the patient may be a candidate for a leadless atrial pacemaker. The potential future development of ventricular pacing dependency also presents a challenge because there is a theoretical risk of ventricular oversensing. However, this can be overcome by adjusting the sensing threshold drop time of the EV-ICD.^5^

## Conclusion

This case demonstrates the feasibility and safety of EV-ICD implantation in a patient with a preexisting atrial pacing–dependent TV-DCPPM system. Successful implantation relies on meticulous preimplantation planning, precise intraoperative lead placement and testing, and careful postimplant EV-ICD programming.

## Disclosures

The authors have no conflicts of interest to disclose. The other authors have no conflicts of interest to disclose.
